# Closed Loop Recycling of Electric Vehicle Batteries to Enable Ultra-high Quality Cathode Powder

**DOI:** 10.1038/s41598-018-38238-3

**Published:** 2019-02-07

**Authors:** Mengyuan Chen, Zhangfeng Zheng, Qiang Wang, Yubin Zhang, Xiaotu Ma, Chao Shen, Dapeng Xu, Jin Liu, Yangtao Liu, Paul Gionet, Ian O’Connor, Leslie Pinnell, Jun Wang, Eric Gratz, Renata Arsenault, Yan Wang

**Affiliations:** 1 Department of Mechanical Engineering, Worcester Polytechnic Institute, Worcester, MA 01609 USA; 2 A123 Systems, 200 West St, Waltham, MA 02451 USA; 3 Battery Resourcers, 54 Rockdale St, Worcester, MA 01606 USA; 4 Energy Storage & Materials Research, Research and Innovation Center, Ford Motor Co., 2101 Village Road, Dearborn, MI 48120 USA

## Abstract

The lithium-ion battery (LIB) recycling market is becoming increasingly important because of the widespread use of LIBs in every aspect of our lives. Mobile devices and electric cars represent the largest application areas for LIBs. Vigorous innovation in these sectors is spurring continuous deployment of LIB powered devices, and consequently more and more LIBs will become waste as they approach end of life. Considering the significant economic and environmental impacts, recycling is not only necessary, but also urgent. The WPI group has successfully developed a closed-loop recycling process, and has previously demonstrated it on a relatively small scale 1 kg spent batteries per experiment. Here, we show that the closed-loop recycling process can be successfully scaled up to 30 kg of spent LIBs from electric vehicle recycling streams, and the recovered cathode powder shows similar (or better) performance to equivalent commercial powder when evaluated in both coin cells and single layer pouch cells. All of these results demonstrate the closed-loop recycling process has great adaptability and can be further developed into industrial scale.

## Introduction

With the development of mobile devices and electric cars, the demand of lithium-ion batteries (LIBs) keeps increasing. The market value of global lithium-ion battery was $29.86 billion in 2017 and estimated to reach $139.36 billion in 2026^1^. Because of the decreasing cost and increasing efficiency of LIBs, the rechargeable battery market is facing a major transformation. Bernatein estimates that LIBs will occupy 70% of the rechargeable battery market by 2025^2^. Accordingly, the amount of end-of-life LIBs will rise significantly, lagging only in time. It is known that some countries use unsustainable ways to deal with battery waste such as incinerating or landfilling. The materials’ value is lost if no suitable recycling process is applied, and thus valuable resources are lost. Considering both the economical and environmental implications, LIBs entering the waste stream require efficient and environmentally friendly recycling processes^3–6^. Favorable economics would encourage collection, and follow the successful effective recycling precedent set by the lead acid industry.

Currently, recycling approaches can be divided into three main types: pyrometallurgical, hydrometallurgical and direct recycling^7^. Pyrometallurgy uses high temperature to smelt valuable metals in spent LIBs, a temperature above 1000 °C is used to form alloys^8^. High use of energy restrains its lab-scale research, however, pyrometallurgy is widely used in industry because of its simplicity and high productivity. Hydrometallurgy employs chemical process to recycle, multi-step treatments including acid–base leaching, solvent extraction, precipitation and ion exchange and electrolysis are involved due to the chemical complexity of LIB itself  ^9–17^. Direct recycling recover different materials by physical processes. With minimal destruction, the recovered material retains its crystal structure and has a good electrochemical performance^18^. Pyrometallurgy, hydrometallurgy and direct recycling processes can be combined together to accommodate different incoming chemistry and expected outcome materials.

Over the past few years, many different recycling approaches and methods have been proposed and studied although much of the research is still in the lab scale phase. Ren *et al*. employed a novel slag system FeO-SiO_2_-Al_2_O_3_ to recover spent batteries^8^. *In situ* recycling was developed by Li *et al*., they used oxygen-free roasting and wet magnetic separation technique to recover spent LiCoO_2_/graphite batteries^19^. Tanong *et al*. tested several leaching reagents – inorganic acids, organic acids, chelating agents and alkaine agents, and found sulfuric acid was the most efficient solution for solubilizing metals from spent batteries^10^. They further optimize the best leaching condition using a three level Box-Behnken design^10^. Zhan *et al*. used froth flotation technique and separated fine battery electrode materials efficiently^20^. Lien concentrated valuable metals and graphite using membrane technologies^21^. Sonoc et al. firstly employed Donnan dialysis with cation exchange membranes and recovered lithium, transition metals^16^. Meng *et al*. proposed an electrochemical cathode-reduction method to leach LiCoO_2_ from spent LIBs and mechanism was revealed by kinetic analysis^17^. Shi *et al*. developed a simple process to regenerate spent LiCoO_2_ cathode, and the resulting cathode had a high electrochemical performance^18^. In addition, a number of research development specifically related to hydrometallurgical technologies in recent years are listed in Table 1. Hydrometallurgical recycling mainly involves leaching, solvent extraction and chemical precipitation. Leaching steps can be divided into alkali leaching and acid leaching, and acid leaching is more favorable because of its higher efficiency. Acid leaching includes inorganic acid and organic acid leaching, and inorganic leaching involves strong acid and can produce secondary pollution, while organic leaching can reach similar efficiency under a milder environment. Another leaching process is bioleaching, and it utilizes the acids generated during microorganisms’ metabolism processes. Inorganic acid leaching has the advantages of low cost while organic acid leaching and bioleaching are more environmentally friendly. Solvent extraction is the process that follows leaching and to separate metal ions or to remove impurities, and it is accomplished because of the various distribution of metal ions between organic solvent and aqueous solution. Due to the high purity of products, solvent extraction is adopted in industry. However, there is still room for improvements to eliminate the complex procedures and high cost of solvent. Chemical precipitation is widely used for separating metals from complex systems due to the varied solubilities at a certain pH. Common precipitants are NaOH, H_2_C_2_O_4_, C_4_H_8_N_2_O_2_, H_3_PO_4_, and Na_2_CO_3_, which can react with transition metal ions or Li^+^ and forms insoluble precipitates. Ni, Mn and Co have similar properties and thus can be co-precipitated as hydroxides, which can be further fabricated into cathode. As such, complex separation steps are avoided and all the values can be recovered. In addition to the primary chemical processes discussed above, other recycling techniques including electrolysis, ion exchange and sol-gel processes are also studied for recycling. However, most of these processes only use single stream of spent batteries for recycling experiments. The produced materials are normally evaluated in the university lab.

**Table 1:** List of hydrometallurgical technologies development in the literature.

Process	Development	Authors and year
Leaching	• Alkali leaching-NH_3_, (NH_4_)_2_SO_4_, Na_2_SO_3_ • Leaching efficiencies of Co, Ni and Li-over 98%	Zheng *et al*.^41^
	• Inorganic acid leaching-HCl • Leaching efficiencies of Co and Mn-over 99%	Barik *et al*.^42^
	• Organic acid leaching-Lactic acid • Leaching efficiencies of Li, Ni, Co and Mn-over 97%	Li *et al*.^43^
	• Bioleaching- organic acids produced by Aspergillus niger • Leaching efficiencies of Cu and Li-100%	Bahaloo-Horeh *et al*.^44^
Solvent extraction	• Solvent extractants-Cyanex 272 and PC-88A • Purities of Li, Ni and Co-99.9%, 99.7% and 99.6%	Virolainen *et al*.^45^
	• Solvent extractants-D2EHPA in kerosene • Purities of Li as Li_2_CO_3_-99.25%	Yang *et al*., 2017^46^
Chemical precipitation	• Precipitants and Precipitates-H_3_PO_4_ and Li_3_PO_4_, H_2_C_2_O_4_ and CoC_2_O_4_ • Recovery efficiencies of Li and Co-88% and 99%	Pinna *et al*.^13^
	• Precipitants and Precipitates-H_3_PO_4_ and Li_3_PO_4_, H_2_C_2_O_4_ and CoC_2_O_4_, C_4_H_8_N_2_O_2_ and Ni(C_4_H_6_N_2_O_2_)2 • Recovery efficiencies of Li, Ni and Co-93%, 96% and 97%	Chen *et al*.^47^
	• Precipitants and Precipitates-NaOH and Ni_x_Mn_y_Co_z_(OH)_2_	Yang *et al*.^48^

Our group has developed a lab-scale and highly efficient closed-loop recycling process previously, which combines hydrometallurgical and direct recycling processes^22–26^. The inorganic acid leaching and co-precipitation reaction in our closed-loop recycling process are the typical hydrometallurgical processes. While different from industrial available hydrometallurgical recycling processes, in which the recovered materials are meal oxides or raw metal alloys, our closed-loop recycling process produced industrial-grade cathode material directly from recycling stream. However, not restricted to the success of recycling in lab scale, this closed-loop recycling process can be transferred into industrial scale and it paved a path to commercially recycle LIBs in a more economic and environmental friendly way. The main and exclusive advantages of our recycling process are as follow: (1) it can accommodate a wide range of incoming LIB chemistry feed. EV batteries from General Motors, Ford and Fiat Chrysler Automobiles were used to demonstrate this flexibility. (2) the recovered cathode material has equal or, in some instances, better electrochemical performance compared with commercial cathode material. (3) the electrochemical results haven been proved by both WPI and A123 Systems independently. (4) the recycling process has been scaled up to 30 kg spent batteries per experiment. Battery Resourcers is further scaling up the recycling work to 0.5ton spent lithium ion batteries per day.

Our previous work has focused primarily on the recycling of electronics battery waste and experiments were conducted up to a maximum scale of 1 kg of battery feed. The properties of recovered cathode materials show good performance; however, they are not as good as the industrial material. Here, with the utilization of the closed-loop recycling process, we report the successful scaling-up of the process with different EV recycling streams and produce very high quality cathode materials-LiNi_1/3_Mn_1/3_Co_1/3_O_2_ (NMC 111). The large scale experiment (7-day) also reveals how the particles are evolved, which has not been reported before. In this work, the end-of-life LIBs come from General Motor Chevrolet Volt (GM), Ford Focus (Ford) and Fiat Chrysler Automobiles 500 e (FCA) vehicles. The batteries used in both GM and Ford’s EV batteries use a cathode which consists of LiNi_1−x−y_Mn_x_Co_y_-LiMn_2_O_4_ (NMC-LMO), while FCA’s battery supplier employs a cathode comprised of LiNi_1−x−y_Mn_x_Co_y_-LiMn_2_O_4_-LiNi_x_Co_y_Al_z_O_2_ (NMC-LMO-NCA)^27–32^. The successful scaling-up of the process was confirmed by our own analyses and independent electrochemical test results from A123 Systems. Compared with the commercially sourced cathode material that was used as our control, our recycled cathode shows comparable electrochemical performances and, notably, superior rate capability.

## Results and Discussions

Parameters optimization of the co-precipitation reaction was accomplished using experiments whose reaction time was 30 hours and in which ~1 kg spent lithium ion batteries were processed. Then parameters were translated to the larger scale experiments, in which 30 kg batteries were used and the reaction time were 168 hours. The ultimate success of the scaling-up of the closed-loop recycling process was verified both by our results and independent electrochemical tests from A123 Systems.

### Small-scale Experiment

In the co-precipitation reaction, parameters that need to be optimized include pH number, residence time, reaction time, the feed rates of the ammonia solution and metal sulfate, etc. Here, metal sulfate solution is added in a range of 2.4–3.5 mL/min, and ammonia water is 0.4–0.9 mL/min. NaOH solution is fed automatically and the pH is controlled in a range of 10–11. In the small-scale experiment with a reaction time of 30 hours, samples are taken every five hours to monitor the process. Every sample is filtered and dried for SEM observation and tap density tests. While the as-filtered sample is pink, it turns into black when dried. It is believed that the black powder is a mixture of hydroxide and oxyhydroxide^33^.

**Figure 1:**
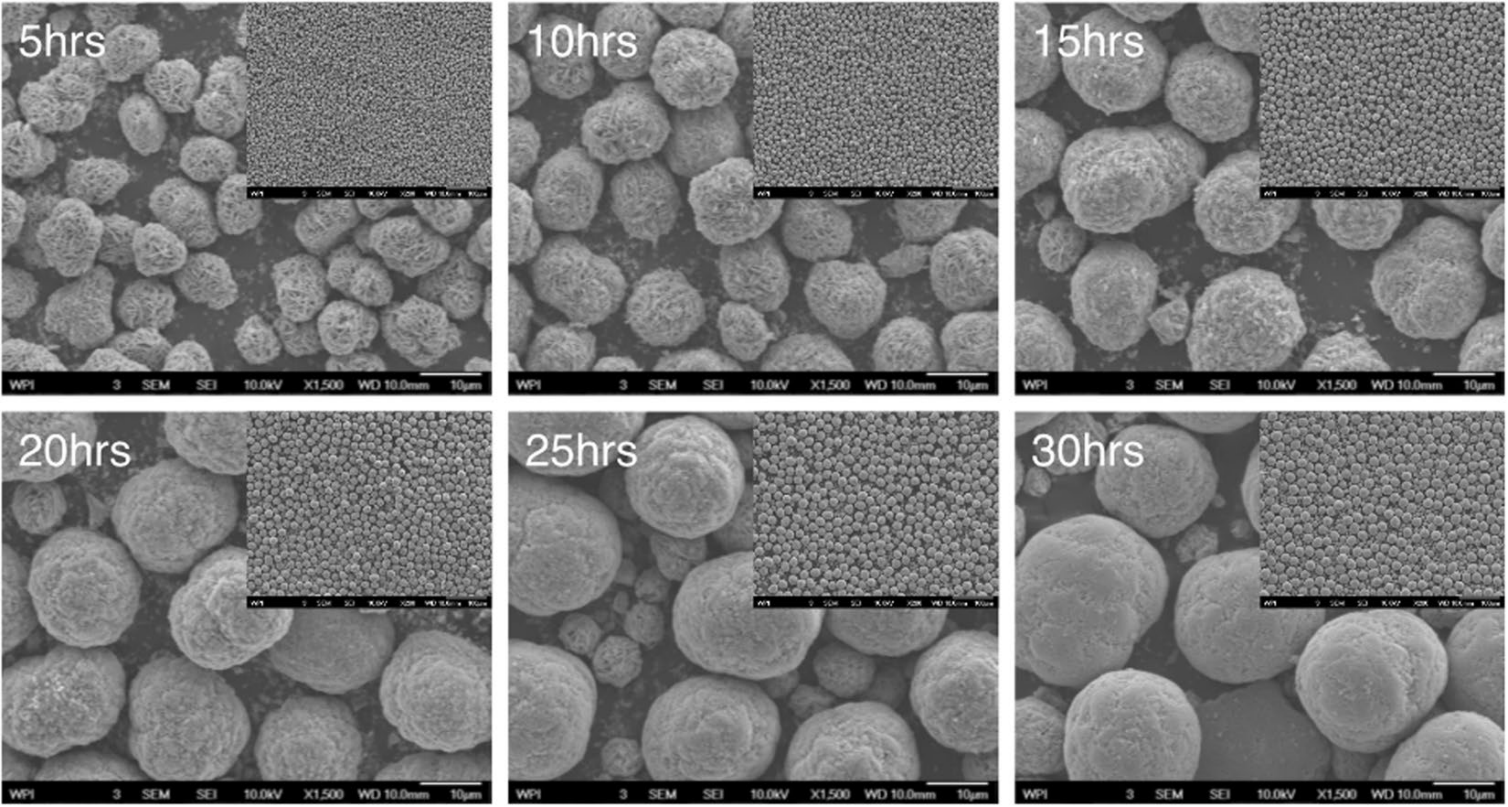
SEM images of precursors that collected at different time. For example, 5 hrs is SEM images of 5 hours precursor with a magnification of 1,500 (scale bar: 10 μm). The top inner right insert shows the SEM images of the 5 hours precursor with a magnification of 200 (scale bar: 100 μm), etc.

Evolution of the precursor particle morphology is observed in the SEM images in Fig. 1, and the tap densities detailed in Supporting Information (Fig. S1). The precursor particles are secondary particles that are spherical in shape, and are aggregates of plate-shaped primary particles. As the reaction progresses, the primary particles keep filling the internal void space available within the secondary particle. Via this mechanism, the precursor particles grow progressively larger and denser. Tap densities in Supporting Information (Fig. S1) supports this trend. At some point, there is no more space for thickening and filling, and particle size achieves steady state and remains constant, beyond which tap density stops increasing significantly. This signals that the co-precipitation reaction has reached equilibrium. In the small-scale experiment, this occurs at 25 hours. Samples collected after 25 hours can be regarded as the same, which laid the foundation for further scale-up and eventual industrial application. In the later scale-up experiments, it was more evident that the reaction reaches an equilibrium after a transient non-steady state period. It is worth mentioning that particle size and morphology play important roles in electrochemical performances. Uniform, spherical particles improve tap density and further boost the capacity/energy density of the electrodes^34^. Smaller particles are beneficial for cell power performance because of shorter ionic diffusion distance. However, the total higher surface area also adversely impacts performance due to increased contact resistance^35^. Optimized particle size distribution benefits both rate tests and cycle performance, and it is believed 10 ± 2 µm of D50 is an ideal range for NMC111 based on the size of commercial NMC 111 powder. Here, the 30-hour precursor sample has a tap density of 2.05 g/mL. The high tap density, uniform morphology and suitable size of the precursor particles make them good candidates for sintering into cathode powder.

Cathode material is fabricated by sintering the final precursor and lithium carbonate. SEM images are included in Supporting Information (Fig. S2), and it can be observed that the cathode powder maintains good morphology and the size of the precursor, which highlights the importance of synthesizing high quality precursor. The tap density of the recycled cathode power is as high as 2.75 g/mL, which is beneficial for specific capacity and electrochemical performance, as will be reported later. The crystalline property and purity of the synthesized cathode are confirmed by XRD and ICP-OES, respectively. The XRD pattern and refinement are presented in Supporting Information (Fig. S2 and Table S1). The peaks of synthesized cathode are sharp and it is classified as α-NaFeO_2_ structure (R$\bar{3}\,$m). Distinct separations of (006)/(012) and (018)/(110) are due to the highly ordered layered structure. The c/3a has a value of 1.6587, which is also an indicator of good layered structure^34^. The XRD pattern difference of synthesized cathode and simulation is marginal, not only from observation but also from the Rwp number (6.50%). The magnitude of ICP-OES tests is in ppm, and both precursor and cathode powder are dissolved and diluted first. Results are shown in Supporting Information (Table S2), and ratios of metal ions are exactly 1:1:1:3 (Ni:Mn:Co:Li) with less than 3% measurement error. Moreover, no impurities are detected in ppm order.

Parameters optimization in small-scale experiments was not only successful in generating high quality cathode material, but offers a valuable tool from which the parameters can be translated to inform larger scale experiments. Cathode material synthesized using our closed-loop recycling process exhibits excellent performances, including tap density, particle morphology and size distribution, crystallization and purity tests (XRD and ICP-OES). It not only demonstrates the viability of our recycling method, but also provides parameters for the following scale-up experiments.

### Scale-up

Herein, the reaction time is increased to seven days compared with thirty hours in the small experiment. For the adjustments in larger scale experiments, we transferred all the parameters into larger scale experiments in their initial status (168 hrs reaction time) after finding the optimized parameters in smaller scale experiments (30 hrs reaction time). However, in order to accommodate to requirements of particle size distribution, tap density, *et al*., parameters including pH number, flow rate, etc would be adjusted during the larger scale experiments while monitoring the experiment closely. Therefore, the parameters in larger scale experiments are not exactly the same with those in smaller experiments.

**Figure 2:**
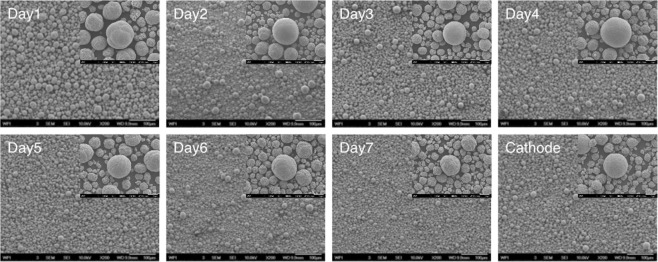
SEM images of precursors that collected at different range of time and the final cathode (bottom right). For example, Day 1 shows SEM images of Day 1 precursor with a magnification of 200 (scale bar: 100 μm). The insert for the Day 1 precursor shows the SEM of the same material with a magnification of 1,500 (scale bar: 10 μm), etc.

Samples are taken every 24 hours and the experiment is monitored to ensure scaled-up experiments are suited to eventual industrial application. It is well known that industrial-scale co-precipitation processes employ continuous reactions. For example, production runs for 30 days are common, with material being collected daily over the course of the run. The key to successful industrialization is to guarantee every batch of power can be used, and thus every day’s precursor is collected for further testing. Tap densities are displayed in Supporting Information (Fig. S3), and they remain constant from day to day, and as high as 2.00 g/mL. Particle size distribution seems to show some differences over the first three days, as observed in the SEM images in Fig. 2. Firstly, the particles have uniform size. With the progress of the experiment, the particles grow larger and new particles are formed. After 3~4 days, the reaction reaches to steady state and at this time the particle size and distribution will not change much with further progress of the experiment. The particles collected after steady state have bimodal size distribution. Although it takes longer to attain equilibrium in large-scale experiment, considering industrialization in the future, three days has marginal influence for a one-month production run. Factors including particle morphology, size distribution, and tap density are monitored and once stable, samples from Day 3 to Day 7 are combined together and sintered into cathode powder. It can be seen by the SEM images presented in Fig. 2, that the cathode particles are densely packed and quite spherical, and a tap density of 2.52 g/mL was achieved. Particle size distribution of the synthesized cathode has been characterized by A123 systems and results are shown in Supporting Information (Fig. S4). Material has a D50 of a value of 11.7 µm and the electrochemical performance is presented in later sections. Although the large-scale experiment in our lab was only carried out for seven consecutive days, it is believed that with a larger reactor and constant supply of reactants, commercialization of our closed-loop recycling process is very promising.

These results demonstrate that our recycling process was successfully scaled up from a spent battery feed of 1 kg to one of 30 kg. The synthesized powder characterization results, including particle size, particle morphology, tap density, purity and crystalline state, and their proximity to results from reference commercial cathode material, suggest that the recycled cathode will deliver comparable electrochemical performance in coin cells and pouch cells. This will be addressed in the next section.

### Electrochemical performance

**Figure 3:**
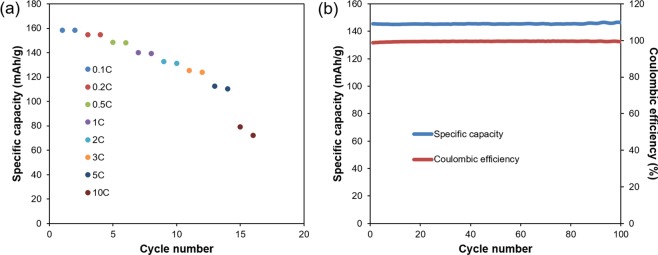
(**a**) Rate performance. (**b**) Cycle performance.

Ultimately the success of our scale-up must be validated by evaluating the electrochemical performance of the recycled cathode material in cells. Accordingly, both half coin cell and full coin cell were assembled in our lab to evaluate rate capability and cycle performance. Results are shown in Fig. 3. Specific capacity is 158 mAh/g, 155 mAh/g, 149 mAh/g, 140 mAh/g, 133 mAh/g, 125 mAh/g, 113 mAh/g, 79 mAh/g for 0.1C, 0.2C, 0.5C, 1C, 2C, 3C, 5C, 10C, respectively. The capacity of 158 mAh/g at 0.1C is comparable with commercial NMC 111 capacity. The high rate performance is quite impressive (113 mAh/g and 79 mAh/g for 5C and 10C, respectively). Besides the excellent rate capability results, the cycle life tests, conducted using 0.5C/0.5C cycling, are also promising. The coulombic efficiency stays above 99% for all of the 100 cycles. After 100 cycles, the capacity retention is nearly 100%. There is a slightly specific capacity increase during the cycling. In general, the slight increase of the specific capacity is due to the following reasons: (1) the increased conductivity of electrode materials. (2) the increase of surface area caused by electrode materials’ minimal breakage. (3) the continuous activation of electrode materials due to better infiltration of electrolyte. (4) the slight increase of room temperature. Here, the increase of specific capacity was very small (1.55 mAh/g, ~1% of the specific capacity of the material) and we believed the slight increase of room temperature maybe the main reason. The coin cells were tested at room temperature, and there was a slight variation of temperature between daytime and nighttime. Also, the continuous stabilization of interface in coin cells and activation of electrode materials may help the slight capacity increase. Compared with commercial NMC 111 powder, our recycled product is comparable, with better rate capability. Considering the aggregate results including SEM image, tap density, size distribution, purity and crystal structure, one can conclude that the closed-loop recycling process described herein has very promising commercial potential.

### Independent Testing at A123 Systems

**Figure 4:**
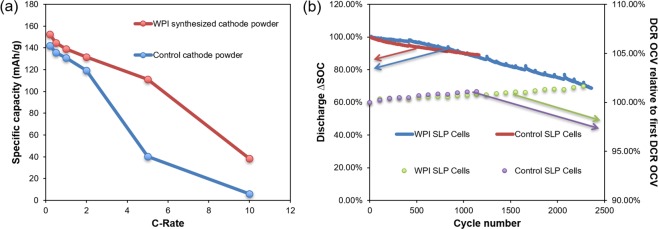
(**a**) Rate performance of coin cells. (**b**) SLP cells cycle performance. (WPI synthesized cathode powder vs. Control cathode powder).

**Figure 5:**
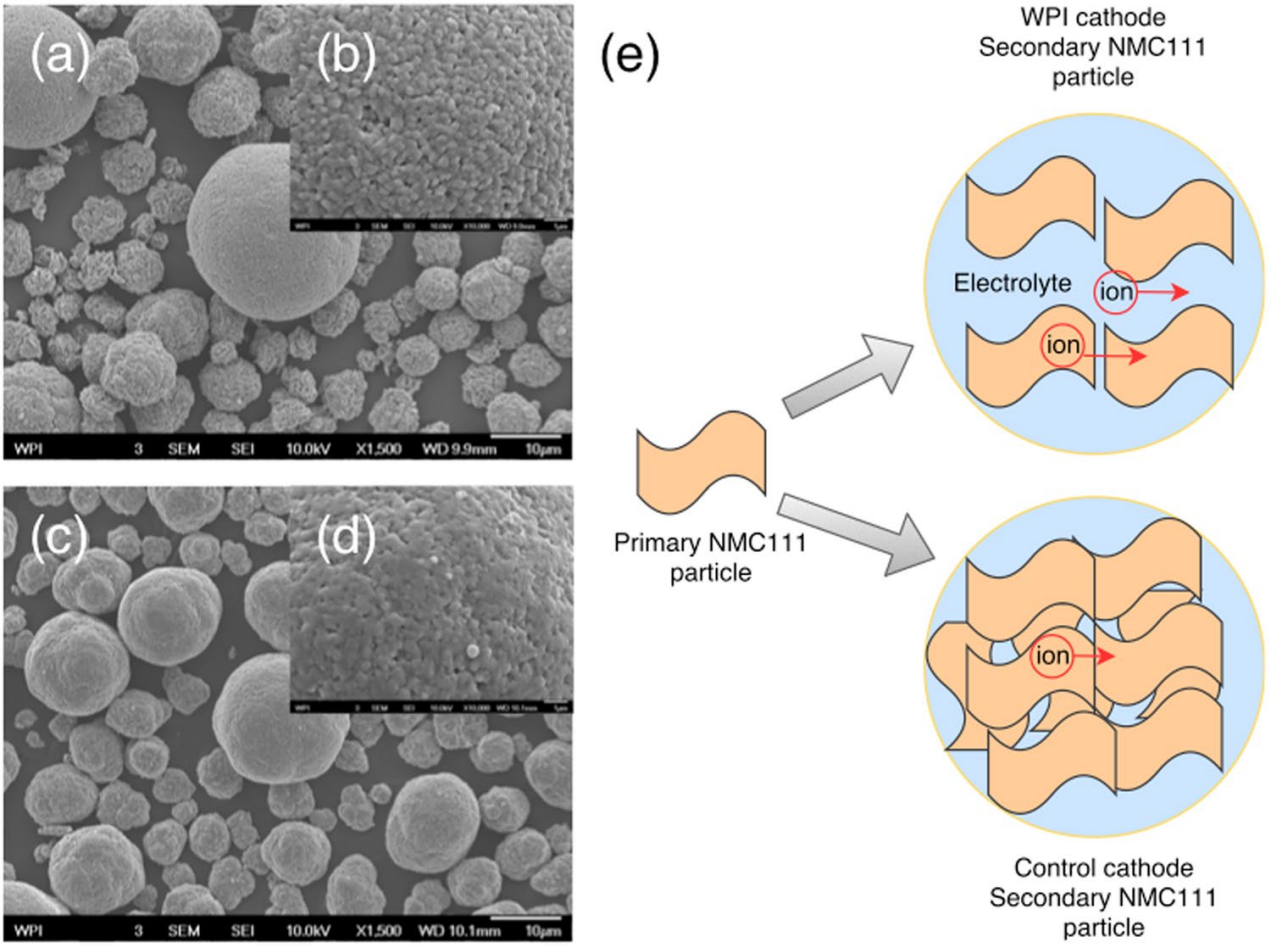
SEM images of cathode powder (WPI vs. Control). (**a**) WPI with magnification factor of 1,500 (scale bar: 10 μm) (**b**) WPI with magnification factor of 10,000 (scale bar: 1 μm) (**c**) Control with magnification factor of 1,500 (scale bar: 10 μm) (**d**) Control with magnification factor of 10,000 (scale bar: 1 μm) (**e**) Schematic demonstration of diffusion difference between WPI synthesized cathode and control cathode.

Synthesized cathode powders from the recycling process were sent to A123 Systems for independent electrochemical performance tests. A123 Systems selected a commercially available NMC111 cathode as a control powder, and compared the control powder with WPI synthesized cathode powder (Fig. 4) in coin cells and single layer pouch cells (SLPs) that were otherwise identical. Detailed comparison of physical properties between WPI synthesized cathode powder with control cathode powder tested by A123 Systems is depicted in Supporting Information (Table S3). It was observed that WPI synthesized powder performed better than the control reference at all rate tested up to 10C (Fig. 4a). A comparison between WPI synthesized cathode powder and commercial control cathode powder is shown in Fig. 5. WPI synthesized cathode is more porous than control cathode, which is especially clear in small particles. Porous cathode particles have more electrolyte uptake and diffusion is easier because ionic diffusion in liquid (diffusion coefficien t ~10^−6^ cm^2^/s)^36^ is faster than that in solid (diffusion coefficient ~10^−10^ cm^2^/s)^37^. It explains that at higher rate, WPI synthesized cathode outperforms control cathode. Specific capacity of WPI synthesized cathode powder has a value 153 mAh/g, 145 mAh/g, 139 mAh/g, 132 mAh/g, 111 mAh/g, 38 mAh/g for 0.2C, 0.5C, 1C, 2C, 5C, 10C, respectively, which is similar to the results in the previous section. Based on those results, the synthesized cathode material from our closed-loop recycling process is well positioned to compete with non-recycled counterparts for Li-ion battery market. Besides coin cell, A123 systems also fabricated single layer pouch (SLP) cell to further study the electrochemical performance of WPI recovered cathode powder.

Pouch cells are widely used in consumer, military and automotive applications because of their simple and lightweight design^38^. The SLP cell is a simple yet representative form factor which largely mimics the multi-layered prismatic cells. Screening battery materials at the SLP level offers a useful research tool for early stage technology development where material quantity may limit the choice of form factors between coin cell and large format pouch cells. Therefore, the electrochemical performance of SLP cells fabricated by A123 Systems has great values and offers insight to inform subsequent work. Results are presented in Fig. 4(b), where control and WPI synthesized cathode show similar trends in both discharge ΔSOC tests and direct current resistance (DCR) tests. After 1000 cycles, 90% of the discharge capacity is retained, and after 1700 cycles, 80% of its discharge capacity remains. Impedance is also comparable for the control and WPI synthesized cathode powders. Independent electrochemical performance tests again yield encouraging results. WPI synthesized cathode powder has comparable or better (for high rates) performance than the commercially sourced control powder. The results suggest that our recycling process is capable of being scaled up. The critical next phase of technology development and large scale validation is presently being conducted by Battery Resourcers, Inc.

## Conclusions

In this study, it is confirmed that our closed-loop recycling process can handle large format spent batteries that come from different commercial electrified vehicles (GM, Ford and FCA), having different cathode chemistries. Moreover, recycled cathode powder synthesized by WPI has similar or better performance when compared to equivalent stoichiometry commercial powder. This is validated by electrochemical testing of WPI assembled coin cells, and independent electrochemical testing of coin cells and SLP cells made and tested by A123 Systems. In addition, this recycling process is scalable (30 kg spent batteries are recycled in each experiment), with efforts towards further scale-up underway. The very promising results obtained to date of this timely and important work suggest a viable path towards commercialization exists. Toward this end, scale-up and development work continues at Battery Resourcers and further results can be expected in the near future.

## Experimental Section

### Overall Recycling Process

In general, the closed-loop recycling stream can be summarized in Supporting Information (Fig. S5). End-of-life batteries are first cut, shredded and sieved. Batteries of various form factors, package design (pouch or metal can) and multiple chemistries can be combined in a single feed lot. After removal of the casing, aluminum etc., what remains is the graphite, carbon and cathode powders. The different cathode powders are dissolved together in a leaching solution of sulfuric acid (H_2_SO_4_) and hydrogen peroxide (H_2_O_2_). In this step, some impurities are also dissolved in the leaching solution. In order to synthesize NMC111, impurities such as Cu, Fe, Al are removed by strategically controlling the pH. Inductively coupled plasma optical emission spectrometry (Perkin Elmer Optima 8000 ICP-OES) is used to determine the concentrations of various metal ions, and nickel sulfate hexahydrate, manganese sulfate monohydrate and cobalt sulfate are added to reach the desired ratio, which is 1:1:1 for Ni, Mn, Co in this study. The co-precipitation reaction will be discussed in detail later. After filtering and drying, precursors (Ni_1/3_Mn_1/3_Co_1/3_(OH)_2_) mix with Li_2_CO_3_. Then the mixture is sintered at 450 °C for 5 hours and 900 °C for 14 hours.

### The Co-precipitation Reaction – Precursor Synthesis

After cutting, shredding and sieving to remove foils and cell case or pouch materials, the remaining powders, which are a mixture of carbon, graphite and cathode powder, are leached in acid. After removing the impurities in the leachate, nickel sulfate hexahydrate (GFS Chemicals), manganese sulfate monohydrate (GFS Chemicals) and cobalt sulfate (GFS Chemicals) are added to adjust the ratio of Ni, Mn, Co. This ratio is tested again using ICP, after which the metal sulfate solution undergoes a co-precipitation reaction. Chemical reagents fed into the co-precipitation reactor are (Ni/Mn/Co) metal sulfate solution, ammonia solution (32%, EMD Millipore) and sodium hydroxide (VWR). A 5 L jacketed glass cylinder with customized feed ports is used for the co-precipitation reaction, which is conducted under nitrogen. Process parameters including pH, flow rate and temperature are controlled throughout the reaction. After a certain transient period, the co-precipitation reaction reaches equilibrium, or steady state, and particle size, morphology and tap density remain constant^39^.

At the end of experiment, the suspension is filtered and washed thoroughly to remove residual or absorbed salts. Then, the particles need to be dried in the oven for about 12 hrs at 130 °C. Tap densities are measured manually, whereby a graduated cylinder is tapped constantly until the level stops changing. JEOL JSM 7000 F is used for obtaining SEM images of the particles. X-ray diffraction (XRD) patterns are obtained using PANalytical Empyrean, with highScore software employed to obtain Rietveld refinement.

### Cathode Sintering

To synthesize cathode active material, 1 mol of precursor is mixed with 1.05 mol of Li_2_CO_3_ (VWR). An excess of 5% Li_2_CO_3_ compensates for the Li loss during firing^40^. Before sintering, the mixture must be uniform in color. Otherwise, it needs to be mixed again. Sintering conditions are 450 °C for 5 hours and 900 °C for 14 hours. Both heating and cooling rates are 2 °C/min. After cooling down to room temperature, the cathode powder must undergo grinding to ensure the material does not contain any agglomerates.

### Electrochemical Testing

Cathode powder, conductive carbon (super C65), and Polyvinylidene fluoride (PVDF) dissolved in N-Methyl-2-pyrrolidone (NMP) are mixed uniformly. Then the slurry is cast onto an aluminum foil and dried at 60 °C for 8 hours. 14 mm diameter electrode disks are punched and pressed to reach a desired porosity prior to assembling into coin cells. After being dried in vacuum oven for 8 hours, any excess solvent is removed, and the cathode electrode is ready for coin cell assembly. In the WPI lab, we assemble half coin cells to test the rate performance and full coin cells to test the cycle performance. Half coin cells use a lithium metal anode while full coin cells use a graphite anode. They are both assembled in glove box. The electrolyte used is 1M LiPF6 in ethylene carbonate (EC), diethyl carbonate (DEC), and dimethyl carbonate (DMC) (1:1:1). The separator used is a 25um trilayer polypropylene-polyethylene-polypropylene membrane (MTI Corporation). Electrochemical performance tests are conducted with an Arbin instrument (Model BT2043). Both coin cells and single layer pouch cells are also assembled at A123 Systems for independent evaluation following similar cell assembly and testing protocols.

## Supplementary information

supporting information

## Data Availability

The dataset generated during the current study are not publicly available due to intellectual property purposes but are available from the corresponding author on reasonable request.
