# Supplementary material for: Closed Loop Recycling of Electric Vehicle Batteries to Enable Ultra-high Quality Cathode Powder
Source: Sci Rep. 2019 Feb 7;9:1654. doi: 10.1038/s41598-018-38238-3 (PMC6367435; doi:10.1038/s41598-018-38238-3)
Supplement: Supplementary file 1 — supporting information [file 41598_2018_38238_MOESM1_ESM.doc]

**Closed Loop Recycling of Electric Vehicle Batteries to Enable Ultra-high Quality** **Cathode Powder**

Mengyuan Chen^1^, Zhangfeng Zheng^1^, Qiang Wang^1^, Yubin Zhang^1^, Xiaotu Ma^1^, Chao Shen^1^, Dapeng Xu^1^, Jin Liu^1^, Yangtao Liu^1^, Paul Gionet^2^, Ian O’Connor^2^, Leslie Pinnell^2^, Jun Wang^2^, Eric Gratz^3^, Renata Arsenault^4^, Yan Wang^1^

1.Department of Mechanical Engineering, Worcester Polytechnic Institute, Worcester, MA 01609, USA

2.A123 Systems, 200 West St, Waltham, MA 02451, USA

3.Battery Resourcers, 54 Rockdale St, Worcester, MA 01606, USA

4.Energy Storage & Materials Research, Research and Innovation Center, Ford Motor Co., 2101 Village Road, Dearborn, MI 48120, USA
E-mail: [yanwang@wpi.edu](mailto:yanwang@wpi.edu)

**Supporting Information**


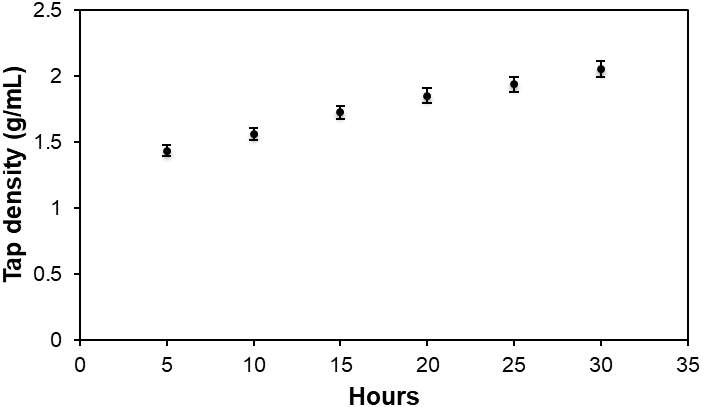


Figure S1: Tap densities of precursors that collected at different time.

Figure S2: XRD pattern and Rietveld refinement of cathode-LiNi_1/3_Mn_1/3_Co_1/3_O_2_. Red line is observed data and blue line is simulation. (a) SEM image of cathode with magnification factor of 1,500 (scale bar: 10 μm) (b) SEM image of cathode with magnification factor of 200 (scale bar: 100 μm)

Table S1: Rietveld Refinement of Synthesized LiNi_1/3_Mn_1/3_Co_1/3_O_2_.

| Lattice parameters | | Agreement indices | | |
| --- | --- | --- | --- | --- |
| a (Å) | c (Å) | Rwp (%) | Rp (%) | Re (%) |
| 2.8613 | 14.2385 | 6.5004 | 5.0411 | 6.5156 |

Table S2: Ratio of metal ions tested by ICP-OES. ND: Not detected

| Ratio | NMC 111 Precursor | NMC 111 Cathode |
| --- | --- | --- |
| Ni | 0.98 | 1.01 |
| Mn | 1.00 | 1.00 |
| Co | 1.00 | 1.00 |
| Li | ND | 3.09 |
| Cu | ND | ND |
| Fe | ND | ND |
| Al | ND | ND |


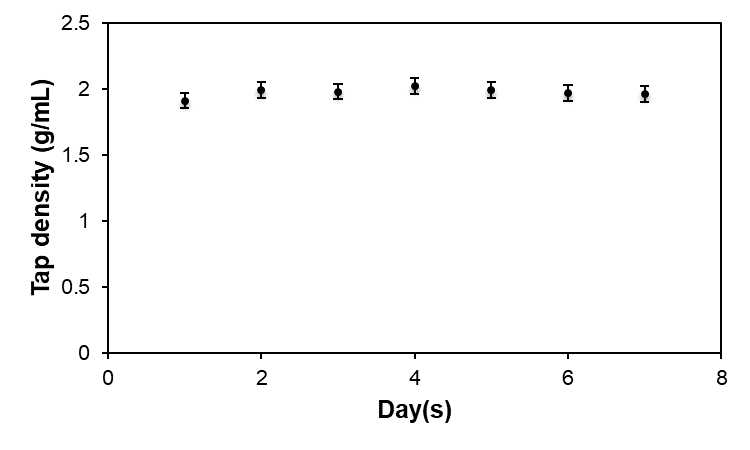


Figure S3: Tap densities of precursors that collected at different time.


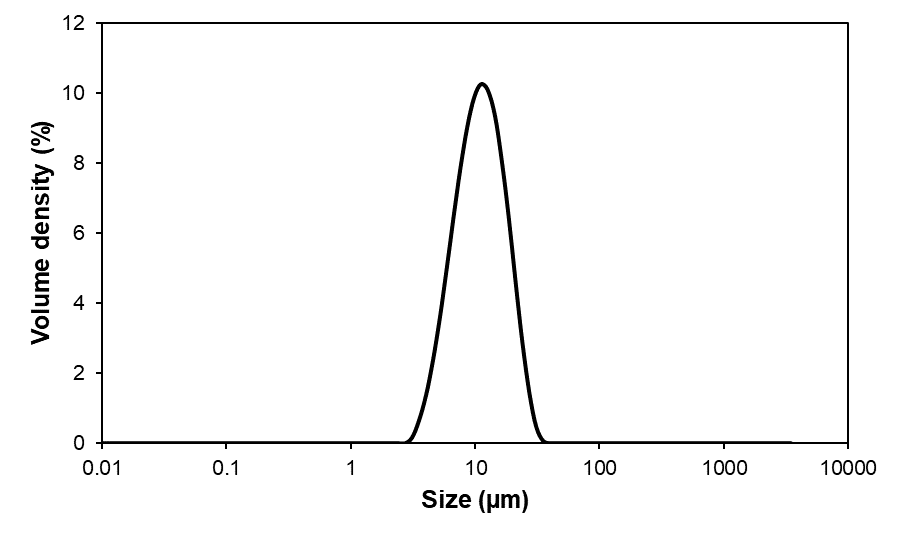


Figure S4: Size distribution of cathode powder. D (10) is 6.22 µm, D (50) is 11.7 µm, D (90) is 21.0 µm, D (99.9) is 35.0 µm.

Table S3: Physical properties comparisons between control cathode powder with WPI synthesized cathode powder.

| Test | Metric | Control Cathode Powder | WPI Synthesized Cathode Powder |
| --- | --- | --- | --- |
| Tap density | g/cc | 2.84 | 2.51 |
| D50 PSD | µm | 9.2 | 10.2 |
| BET | m^2^/g | 0.28 | 0.65 |

Figure S5: Scheme of closed-loop recycling process
